# Imbalanced distribution of regulatory T cells and Th17.1 cells in the peripheral blood and BALF of sarcoidosis patients: relationship to disease activity and the fibrotic radiographic phenotype

**DOI:** 10.3389/fimmu.2023.1185443

**Published:** 2023-07-13

**Authors:** Hui Zhang, Dingyuan Jiang, Lili Zhu, Guowu Zhou, Bingbing Xie, Ye Cui, Ulrich Costabel, Huaping Dai

**Affiliations:** ^1^ Department of Pulmonary and Critical Care Medicine, China-Japan Friendship Hospital, National Center for Respiratory Medicine; National Clinical Research Center for Respiratory Diseases, Institute of Respiratory Medicine, Chinese Academy of Medical Sciences Peking Union Medical University, Beijing, China; ^2^ Department of Pulmonary and Critical Care Medicine, Hunan Provincial People’s Hospital, The First Affiliated Hospital of Hunan Normal University, Changsha, China; ^3^ Department of Immunology, School of Basic Medical Sciences, Capital Medical University, Beijing, China; ^4^ Center for Interstitial and Rare Lung Diseases, Pneumology Department, Ruhrlandklinik, University Hospital, University of Duisburg-Essen, Essen, Germany

**Keywords:** sarcoidosis, regulatory T cells, Th17.1 cells, peripheral blood, bronchoalveolar lavage fluid

## Abstract

**Rationale:**

Sarcoidosis is a granulomatous interstitial lung disease involving a complex interplay among different cluster of differentiation 4 (CD4^+^) thymus cell (T-cell) subsets. Originally described as a type 1 T-helper (Th1) inflammatory disease, recent evidence suggests that both effector and regulatory T-cell subgroups play a critical role in sarcoidosis, but this remains controversial.

**Objectives:**

We aimed to investigate the distribution of CD4^+^ T-cell subpopulations in sarcoidosis patients and its potential associations with clinical disease activity and a radiographic fibrotic phenotype.

**Methods:**

We measured the frequencies of regulatory T cells (Tregs), Th1, Th17, and Th17.1 cells in the peripheral blood and/or bronchoalveolar lavage fluid (BALF) of 62 sarcoidosis patients, 66 idiopathic pulmonary fibrosis (IPF) patients, and 41 healthy volunteers using flow cytometry. We also measured the changes in these T-cell subpopulations in the blood at the follow-up visits of 11 sarcoidosis patients.

**Measurements and results:**

An increased percentage of Tregs was observed in the peripheral blood of sarcoidosis patients, with a positive association to disease activity and a fibrotic radiographic phenotype. We found a higher frequency of Tregs, a lower proportion of Th17.1 cells, and a lower ratio of Th17.1 cells to total Tregs in the peripheral blood of both active and fibrotic sarcoidosis patients, compared with IPF patients or healthy donors. In contrast, a lower frequency of Tregs and a higher proportion of Th17.1 cells was found in the BALF of sarcoidosis patients than in that of IPF patients. There was an imbalance of Tregs and Th17.1 cells between the peripheral blood and BALF in sarcoidosis patients. Following immunoregulatory therapy, the proportion of circulating Tregs in sarcoidosis patients decreased.

**Conclusion:**

A higher proportion of Tregs in the peripheral blood of sarcoidosis patients was related to disease activity, fibrotic phenotype, and the need for immunoregulatory therapy. The imbalanced distribution of Tregs and Th17.1 cells in patients’ peripheral blood and BALF suggests that the lung microenvironment has an effect on the immunological pathogenesis of sarcoidosis. Therefore, further studies on the functional analysis of Tregs and Th17.1 cells in sarcoidosis patients are warranted.

## Introduction

Sarcoidosis is a multisystemic inflammatory disorder of unknown etiology, which is characterized by the presence of non-caseating granulomas, and predominantly affects the lungs (1). Patients with sarcoidosis show a wide range of clinical presentations, natural disease course, and disease outcome; this can be attributed to individuals’ genetic predisposition and environmental factors (2). The majority of sarcoidosis patients reach remission with or even without treatment, whereas a minority develop chronic and sometimes progressive disease, even leading to death (3). However, until now, no single biomarker has been identified as being useful in establishing a definite diagnosis of sarcoidosis and/or predicting the disease outcome (4, 5). The exact pathogenesis of sarcoidosis remains incompletely understood, but a cluster of differentiation 4 (CD4^+^) T-cell-induced immune response is generally acknowledged to be a key player in the maintenance of the granulomatous inflammation (6, 7).

Recent advances in better understanding the function of diverse immune cells in sarcoidosis suggest, besides CD4^+^ T cells, a critical role of effector and regulatory T-cell subgroups in the pathogenesis of this disorder (8). This complements the original concept of sarcoidosis as a disease driven by a Th1-dominant immune response, with abundant cytokine expression of interferon-gamma (IFN-γ), interleukin (IL)-2, IL-12, and IL-18 (9–12). Emerging evidence indicates that Th17 cells associated with enhanced IL-17A expression play an important role in sarcoidosis, as they do in many other autoimmune diseases (13–15). A subset of Th17 cells, called Th17.1 cells, is characterized by its capacity to produce both IL-17 and IFN-γ, apparently combining the functional properties of Th17 cells and Th1 cells (16–18). It is still unclear, however, whether Th17.1 cells play a protective or pathogenic role in sarcoidosis. In regard to their association with a favorable prognosis or a chronic disease course, two studies have reported discordant results (19, 20). Reports on the role of regulatory T cells (Tregs) in sarcoidosis are also conflicting, with the cells being reported as having both increased and decreased frequencies (21–23). Furthermore, an elevated percentage of Tregs, but with an impaired immunosuppressive capacity, has also been observed in sarcoidosis patients (24, 25). Intriguingly, it has also been postulated that an imbalance of Th17 cells and Tregs is responsible for disease progression (26).

In this study, we aimed to investigate the distribution of CD4^+^ effector and regulatory T-cell subpopulations in sarcoidosis patients in more detail and analyze their association with disease activity and radiographic phenotype. For this purpose, we used flow cytometry to measure the levels of Tregs, Th1, Th17, and Th17.1 cells in the peripheral blood and/or the bronchoalveolar lavage fluid (BALF) of patients with sarcoidosis or idiopathic pulmonary fibrosis (IPF), and of healthy volunteers.

## Materials and methods

### Study design and participants

We consecutively enrolled 62 participants with sarcoidosis who had been diagnosed at the Department of Pulmonary and Critical Care Medicine, China-Japan Friendship Hospital, from January 2019 to January 2021. The diagnosis of sarcoidosis was confirmed through multidisciplinary discussion (MDD), based on compatible clinical and chest CT findings, together with the presence of non-necrotizing granulomatous inflammation, and the exclusion of alternative causes of granulomatous disease, consistent with the statement from the American Thoracic Society (ATS)/European Respiratory Society (ERS)/World Association of Sarcoidosis and Other Granulomatous Disorders (WASOG) (1). Exclusion criteria were acute respiratory tract infection, concomitant pulmonary disease [including chronic obstructive pulmonary disease (COPD) and asthma], autoimmune diseases, malignancies, organ transplantation, and allergies. The disease activity was defined as follows: (a) recently developed or increasing symptoms such as cough, dyspnea, weakness, fever, and arthralgia; and/or (b) evidence of progressive disease on chest CT; and/or (c) test results indicating the deterioration of lung function; and/or (d) abnormal laboratory test results, such as high levels of serum angiotensin-converting enzyme (sACE), and lymphocytosis with an increased ratio of CD4^+^/CD8^+^ cells in BALF (1, 27, 28). The study design did not require individuals to be newly diagnosed or treatment naive. The treatments given included glucocorticoids, methotrexate, azathioprine, mycophenolate, and biological agents. Follow-up visits were conducted 3 months after enrollment in the study.

The disease control group consisted of 66 individuals who had been diagnosed with IPF. All participants met the international diagnostic criteria for IPF; for this group, we used the same exclusion criteria that had been used for the sarcoidosis patient group (29).

The 41 healthy individuals underwent health check-ups, wherein no chest radiographic abnormalities and no history of malignancies, autoimmune diseases, allergies, or other lung disorders were identified.

The Research Review Committee and the Ethical Review Committee of the China-Japan Friendship Hospital approved this study. Every participant provided written informed consent to participate in the study.

### Peripheral blood mononuclear cells and bronchoalveolar lavage

The fasting peripheral blood was taken within 2 days of bronchoalveolar lavage (BAL) being carried out for sarcoidosis and IPF patients. For those patients who did not undergo bronchoscopy at the baseline or at follow-up visits, and for healthy volunteers, fasting blood was drawn. Whole blood was then collected in sodium heparin tubes. Peripheral blood mononuclear cells (PBMCs) were then isolated using Ficoll-Paque™ Plus (GE Healthcare) density gradient separation followed by washing twice with phosphate-buffered saline (PBS; Hyclone), as previously described (30).

BAL was conducted during bronchoscopy after the administration of local anesthesia and before tissue biopsies, as previously described (31). The choice of the lavage site was guided by the location of the parenchymal pathology on high-resolution computed tomography (HRCT), and, if it was diffuse, the right middle lobe was chosen. With the bronchoscope in a wedged position in a segmental bronchus, 100 mL of sterile saline solution (0.9% NaCl) was decanted into aliquots of 30 mL, 30 mL, 40 mL, and another 40 mL was decanted if necessary. BALF was retrieved by applying a gentle suction into a silicone-treated bottle, and was stored at 4°C until processing within 4 hours of collection. BALF was filtered through a 40-*μ*m cell strainer (BD Biosciences) and centrifuged. Erythrocytes were eliminated using lysing buffer (BD Biosciences) and washing with PBS.

Both PBMCs and BAL cells were frozen in 1 mL of fetal bovine serum (FBS; Gibco) with 10% dimethyl sulfoxide (DMSO; Sigma) in a cryovial using a 5100 Cryo 1°C Freezing Container (Nalgene) to 80°C, and, thereafter, stored in a liquid nitrogen biorepository.

### Flow cytometry

**Figure 1:**
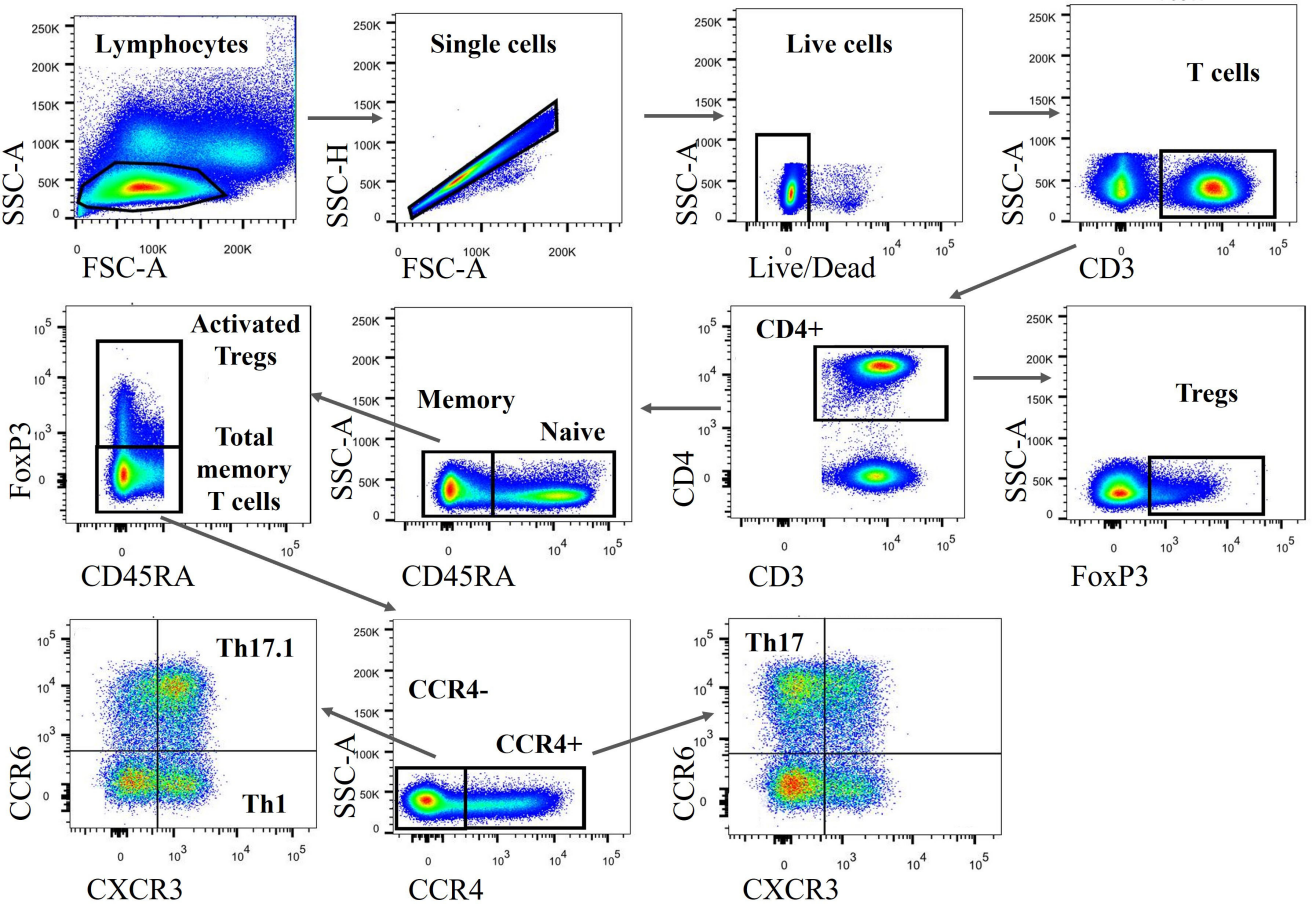
Gating strategy to identify various CD4^+^ T-cell subsets. Both PBMCs and BAL cells were subsequently gated on lymphocytes (based on FSC-A vs. SSC-A), single cells (based on FSC-A vs. SSC-H), live cells (defined as Fixable Viability Stain 510 negatively stained cells), CD3^+^, and CD4^+^ cells. Subsequently, FOXP3^+^ Tregs, FOXP3^+^ CD45RA- activated Tregs and FOXP3^–^ CD45RA^–^ total memory T cells were identified. Stepwise, we classified those total memory T cells into different subgroups, including Th1, Th17, and Th17.1 cells based on the expression of *CCR4*, *CXCR3*, and *CCR6*. PBMCs, peripheral blood mononuclear cells; BAL, bronchoalveolar lavage; CD4^+^, cluster of differentiation 4; Th1, T helper type 1; FSC-A, forward scatter area; SSC-A, side scatter area; SSC-H, side scatter height; CXCR3, chemokine (C-X-C motif) receptor 3; CXR4, chemokine (C-C motif) receptor 4..

PBMCs and BAL cells were analyzed using an LSRFortessa™ flow cytometer (BD Biosciences) by employing an eight-parameter flow cytometry panel. Frozen cells were thawed, resuspended in PBS, and stained on ice in the dark. Staining with Fixable Viability Stain 510 (BD Horizon™) at a 1 : 1,000 dilution in PBS was carried out for the assessment of cell viability and staining with Fc Block™ (BD Pharmingen™) was conducted to prevent non-specific staining. For surface staining, we incubated cells in Brilliant Stain Buffer (BD Horizon) with antibodies per the manufacturer’s instructions, including CD3 PerCP-eFluor 710 (Invitrogen), CD4 BUV395 (BD Horizon), CD45RA BV605 (BD Horizon), CXCR3 (CD183) BV786 (BD OptiBuild™), CCR4 (CD194) PE-CF594 (BD Horizon), and CCR6 (CD196) BB515 (BD Horizon). For intracellular staining, we used the forkhead box protein P3 (FOXP3) Alexa Fluor^®^ 647 antibody (BD Pharmingen), in addition to the Transcription Factor Buffer Set (BD Pharmingen) for both fixation and permeabilization. The flow cytometry characteristic definitions of Tregs, Th1, Th17, and Th17.1 cells are listed in **Table 1**. FlowJo v10.7 software (BD) was used to analyze the flow cytometric data and the gating strategy is shown in **Figure 1**.

**Table 1:** CD4^+^ T-cell subset phenotypes.

CD4^+^ T-cell subset	FOXP3	CD45RA	*CXCR3*	*CCR4*	*CCR6*
Tregs	**+**	**/**	**/**	**/**	**/**
Th1	**−**	**−**	**+**	**−**	**−**
Th17	**−**	**−**	**−**	**+**	**+**
Th17.1	**−**	**−**	**+**	**−**	**+**

Th1, T helper type 1; Tregs, regulatory T cells. +, positively stained; **−**, negatively stained;/, no matter how the subsequent staining.

### Statistical analysis

SPSS Statistics version 16.0 (IBM Corporation, Armonk, NY, USA) and GraphPad Prism 8.0 (GraphPad Software Inc., CA, USA) were used. Comparisons between the groups were assessed by way of an unpaired Student’s *t*-test, the Mann–Whitney *U*-test, chi-squared analysis, or a one-way ANOVA test, as appropriate. A paired Student’s *t*-test was used for the paired comparison. Pearson or Spearman rank correlation tests were used to investigate correlations. A *p*-value of less than 0.05 was considered to be statistically significant.

## Results

### Characteristics of participants

There were 62 patients with sarcoidosis, 66 patients with IPF, and 41 healthy volunteers included in this study (**Table 2**). The mean age of sarcoidosis patients was 52 years; this was lower than that of IPF patients, but not different from that of the healthy donors. Sarcoidosis patients were predominantly female and non-smokers. Sarcoidosis patients had a higher proportion of lymphocytes and a higher ratio of CD4^+^/CD8^+^ cells in their BALF compared with IPF patients. There were 25 patients in the active sarcoidosis group, and 37 in the inactive group. Nearly one-third (20/62) of sarcoidosis patients had extrapulmonary organ involvement, including peripheral lymph nodes, skin, eyes and others.

**Table 2:** Demographics and clinical characteristics.

Characteristic	Sar (*n* = 62)	IPF (*n* = 66)	HC (*n* = 41)
Age (years)	52 ± 13	66 ± 8 ^∏^	48 ± 9
Male/female	15/47	59/7	7/34
Current smoker/former smoker/never smoked	6/5/51	8/40/18	4/2/35
Sarcoidosis stage I/II/III/IV	7/43/7/5		
Sarcoidosis active/inactive	25/37		
Sarcoidosis fibrotic/non-fibrotic	12/50		
Airway mucous nodule	20		
Extrapulmonary organ involvement	20		
Immunoregulatory medication	17		
sACE (U/L)	47.3 ± 31.2		
CD4/CD8 ratio in BALF	6.6 ± 8.2	1.0 ± 0.7 ^∏^	
Lymphocytes in BALF(%)	34.8 ± 39.8	9.0 ± 10.2 ^∏^	
FEV_1_%/pre	92.4 ± 20.8	82.2 ± 18.3 ^∏^	
FVC%/pre	103.3 ± 20.6	78.8 ± 18.5 ^∏^	
TLC%/pre	90.3 ± 15.6	68.2 ± 14.7 ^∏^	
DLCO_SB_%/pre	81.9 ± 19.3	50.1 ± 18.2 ^∏^	
6MWD/m	499.9 ± 69.7	468.3 ± 89.6	

Data are shown as mean ± SEM or number.

Chest radiography staging was based on the Scadding stage criteria.

**^∏^**: statistic difference (p < 0.05) between sarcoidosis and IPF patients.

Sar, sarcoidosis; IPF, idiopathic pulmonary fibrosis; HC, healthy control; sACE, serum angiotensin-converting enzyme; BALF, bronchoalveolar lavage fluid; FEV_1_, forced expiratory volume in 1 second; FVC, forced vital capacity; TLC, total lung capacity; DLCO, carbon monoxide diffusing capacity; 6MWD, 6-minute-walk distance.

### The proportion of circulating Tregs in sarcoidosis patients is associated with disease activity and fibrotic radiographic stages

**Figure 2:**
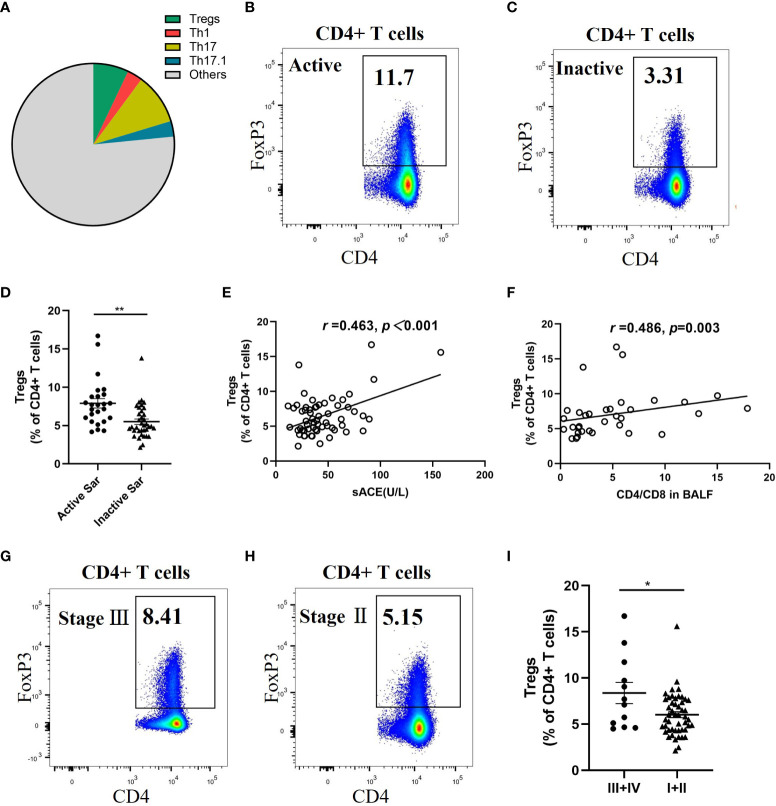
Significant increase of circulating Tregs proportion in active and fibrotic sarcoidosis patients. **(A)** Pie chart showing the mean percentage of Tregs, Th1, Th17, Th17.1, and other unmentioned CD4^+^ T cells in the peripheral blood of sarcoidosis patients. **(B, C)**. Flow cytometry dot plot of Tregs gated by the expression of FOXP3 from an active and an inactive sarcoidosis patient, respectively. **(D)** Comparison of the proportion of circulating Tregs between active and inactive sarcoidosis patients (7.9% ± 1.5% vs. 5.5% ± 0.4%; *p = *0.001). **(E, F)** Positive correlations between the proportion of Tregs and the sACE level (*n* = 60) and the ratio of CD4^+^/CD8^+^ cells in BALF (*n* = 34) of sarcoidosis patients (*r* = 0.463, *p *< 0.001; *r* = 0.486, *p *= 0.003). **(G, H)** Flow cytometry dot plot of Tregs from a stage III and a stage II sarcoidosis patient, respectively. **(I)** Comparison of the proportion of circulating Tregs between fibrotic (stage III/IV) and non-fibrotic (stage I/II) sarcoidosis patients (8.4% ± 3.1% vs. 6.0% ± 0.4%; *p *= 0.045). Data are expressed as mean ± SEM. An unpaired Student’s *t*-test was used to compare the proportion of circulating Tregs in active sarcoidosis patients with that in inactive sarcoidosis patients, and that in fibrotic patients with that in non-fibrotic sarcoidosis patients. Pearson and Spearman rank correlation tests were used to investigate correlation. *p < 0.05; **p < 0.01. BALF, bronchoalveolar lavage fluid; CD4^+^, cluster of differentiation 4; sACE, serum angiotensin converting enzyme; Th1, T helper type 1; Tregs, regulatory T cells.

The flow cytometric analysis of the proportion of diverse CD4^+^ T-cell subsets in the blood of sarcoidosis patients showed that Tregs, Th1, Th17, and Th17.1 cells constituted 7.1% ± 0.7%, 3.0% ± 0.3%, 10.25% ± 0.6%, and 3.15% ± 0.3%, of CD4+ T cells, respectively (**Figure 2A**). The proportion of Tregs in patients with active sarcoidosis was higher than in those with inactive sarcoidosis (7.95% ± 1.5% vs. 5.55% ± 0.4% of CD4^+^ T cells; *p *= 0.001) (**Figures 2B–D**). The proportion of Tregs correlated positively with levels of sACE and the ratio of CD4^+^/CD8^+^ cells in patients’ BALF (*r* = 0.463, *p *< 0.001; and *r* = 0.486, *p *= 0.003) (**Figures 2E, F**). Patients with fibrotic radiographic stages had a higher proportion of Tregs than those with non-fibrotic stages (8.4% ± 3.1% vs. 6.05% ± 0.4% of CD4^+^ T cells; *p *= 0.045) (**Figures 2G–I**).

### Higher percentage of circulating Tregs and lower proportion of Th17.1 cells in active and fibrotic sarcoidosis patients

**Figure 3:**
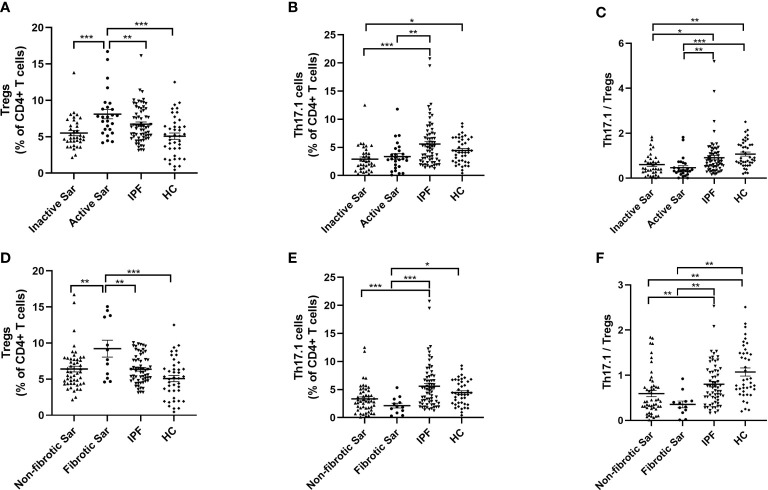
Comparative analysis of the distribution of Tregs and Th17.1 cells in the peripheral blood of sarcoidosis and IPF patients and healthy volunteers. **(A)** The proportion of circulating Tregs of active sarcoidosis patients ranked first, rather than that of inactive sarcoidosis or IPF or HC subjects (7.9% ± 1.5% vs. 5.5% ± 0.4%, vs. 6.7% ± 0.3%, vs. 5.1% ± 0.4%; *p *< 0.001, *p *= 0.004, and *p *< 0.001). **(B)** Both active and inactive sarcoidosis patients had a lower proportion of Th17.1 cells than IPF patients (3.4% ± 0.5% and 2.9% ± 0.4% vs. 5.6% ± 0.5%; *p *= 0.002 and *p *< 0.001). The percentage of Th17.1 cells was also lower in inactive sarcoidosis patients than in healthy volunteers (4.4% ± 0.3%; *p*=0.031). **(C)** The ratio of Th17.1 cells to total Tregs in both active and inactive sarcoidosis patients was lower than that in IPF patients or healthy donors (0.5 ± 0.1 and 0.6 ± 0.1 vs. 0.9 ± 0.1 and 1.1 ± 0.1; *p *= 0.003 and *p *< 0.001; *p *= 0.021 and *p *= 0.002). **(D)** The proportion of Tregs in fibrotic sarcoidosis patients was higher than that in non-fibrotic sarcoidosis, IPF, or HC subjects (8.4% ± 3.1% vs. 6.0% ± 0.4%, vs. 6.4% ± 0.3%, vs. 5.1% ± 0.4%; *p *= 0.005, *p *= 0.009, and *p *< 0.001). **(E)** Both fibrotic and non-fibrotic sarcoidosis patients had a lower proportion of Th17.1 cells than IPF patients (2.1% ± 0.4% vs. 3.3% ± 0.4% and 5.6% ± 0.5%; *p*<0.001 and *p*<0.001). The percentage of Th17.1 cells was also lower in fibrotic sarcoidosis patients than that in healthy donors (4.4% ± 0.3%; *p*=0.023). **(F)** The ratio of Th17.1 cells to total Tregs in both fibrotic and non-fibrotic sarcoidosis patients was lower than that in patients with IPF or HC (0.4 ± 0.1 and 0.6 ± 0.1 vs. 0.9 ± 0.1 and 1.1 ± 0.6; *p* = 0.006 and *p* = 0.001; *p* = 0.009 and *p* = 0.001). One-way ANOVA test was used for data analysis. *p < 0.05; **p < 0.01; ***p <0.001. Sar, sarcoidosis; IPF, idiopathic pulmonary fibrosis; HC, healthy control; Tregs, regulatory T cells; Th17.1, T helper type 17.1.

We found an increased proportion of circulating Tregs in active sarcoidosis patients compared with patients with inactive sarcoidosis or IPF or healthy volunteers (7.95% ± 1.5% vs. 5.5% ± 0.4% vs. 6.7% ± 0.3% vs. 5.1% ± 0.4%; *p *< 0.001, *p *= 0.004, and *p *< 0.001, respectively) (**Figure 3A**). We found that both active and inactive sarcoidosis patients had lower proportions of Th17.1 cells than IPF patients (3.4% ± 0.5% and 2.9% ± 0.4% vs. 5.6% ± 0.5%; *p *= 0.002 and *p *< 0.001, respectively) (**Figure 3B**). The percentage of Th17.1 cells was also lower in inactive sarcoidosis patients than in healthy donors (4.4% ± 0.3%; *p *= 0.031) (**Figure 3B**). Moreover, the ratio of Th17.1 cells to total Tregs was lower for both active and inactive sarcoidosis patients than for IPF patients or healthy volunteers (0.5 ± 0.1 and 0.6 ± 0.1 vs. 0.9 ± 0.1 and 1.1 ± 0.1; *p *= 0.003 and *p *< 0.001; *p *= 0.021 and *p *= 0.002, respectively) (**Figure 3C**). Likewise, the proportion of Tregs was higher in fibrotic sarcoidosis patients than in non-fibrotic sarcoidosis, IPF patients, or healthy participants (8.4% ± 3.1% vs. 6.0% ± 0.4%, vs. 6.4% ± 0.3%, vs. 5.1% ± 0.4%; *p *=* *0.005, *p *= 0.009, and *p *< 0.001, respectively) (**Figure 3D**). However, the percentage of Th17.1 cells for both fibrotic and non-fibrotic sarcoidosis patients was lower than that of IPF patients (2.1% ± 0.4% vs. 3.3% ± 0.4% and vs. 5.6% ± 0.5%; *p *< 0.001 and *p *< 0.001, respectively) (**Figure 3E**). We found that there was a lower proportion of Th17.1 cells in fibrotic sarcoidosis patients than in healthy controls (4.4% ± 0.3%; *p *= 0.023) (**Figure 3E**). Consequently, the ratio of Th17.1 cells to total Tregs was decreased for not only fibrotic but also non-fibrotic sarcoidosis patients, compared with IPF patients or healthy individuals (0.4 ± 0.1 and 0.6 ± 0.1 vs. 0.9 ± 0.1 and 1.1 ± 0.6; *p *= 0.006 and *p *= 0.001; *p *= 0.009 and *p *= 0.001, respectively) (**Figure 3F**).

### Decreased percentage of Tregs and increased proportion of Th17.1 cells in BALF from sarcoidosis patients compared with IPF patients

**Figure 4:**
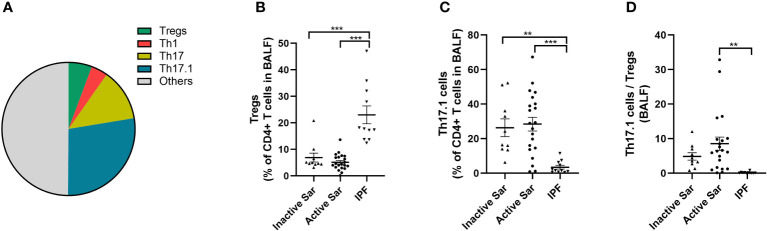
Comparative analysis of the distribution of Tregs and Th17.1 cells in BALF among active or inactive sarcoidosis and IPF patients. **(A)** Pie chart showing the mean percentage of Tregs, Th1, Th17, Th17.1, and other unmentioned CD4^+^ T cells in the BALF of sarcoidosis patients. **(B)** We found a lower proportion of Tregs in the BALF of both active and inactive sarcoidosis patients than in that of IPF patients (5.2% ± 0.7% and 6.9% ± 1.7% vs. 23.0% ± 3.4%; *p *< 0.001 and *p *< 0.001). **(C)** The percentage of BALF Th17.1 cells in both active and inactive sarcoidosis patients was higher than that in IPF patients (28.4% ± 4.0% and 26.3% ± 5.1% vs. 3.4% ± 1.1%; *p *< 0.001 and *p *= 0.001). **(D)** The ratio of Th17.1 cells to total Tregs in the BALF of active sarcoidosis patients was significantly higher than that in IPF patients (8.5 ± 2.4 vs. 0.2 ± 0.1; *p *= 0.004). One-way ANOVA test was used for data analysis. **p < 0.01; ***p<0.001. BALF, bronchoalveolar lavage fluid; Sar, sarcoidosis; IPF, idiopathic pulmonary fibrosis; Tregs, regulatory T cells; Th17.1, T helper type 17.1.

Tregs, Th1, Th17, and Th17.1 cells constituted 5.7% ± 0.7%, 4.0% ± 0.6%, 12.7% ± 2.6%, and 27.7% ± 3.1% of CD4^+^ T cells in the BALF of sarcoidosis patients, respectively (**Figure 4A**). The percentage of Tregs in the BALF of both active and inactive sarcoidosis patients was significantly lower than in IPF patients (5.2% ± 0.7% and 6.9% ± 1.7% vs. 23.0% ± 3.4%; *p *< 0.001 and *p *< 0.001, respectively) (**Figure 4B**). In addition, the percentage of Th17.1 cells was significantly higher than in IPF patients (28.4% ± 4.0% and 26.3% ± 5.1% vs. 3.4% ± 1.1%; *p *< 0.001 and *p *= 0.001, respectively) (**Figure 4C**). Consequently, the ratio of Th17.1 cells to total Tregs in BALF was higher in patients with active sarcoidosis than in those with IPF (8.5 ± 2.4 vs. 0.2 ± 0.1; *p *= 0.004) (**Figure 4D**).

### Imbalanced distribution of Tregs and Th17.1 cells in the peripheral blood and BALF of sarcoidosis patients

**Figure 5:**
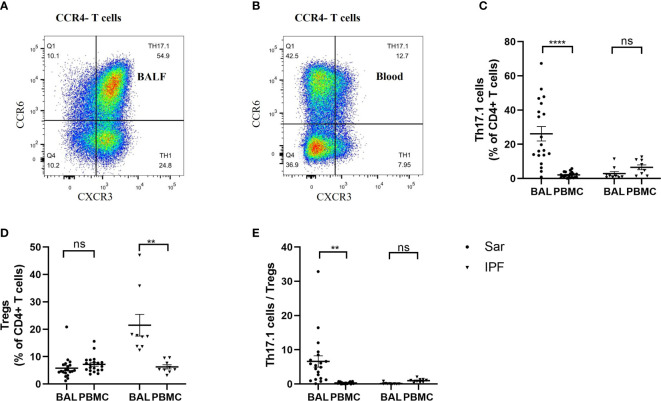
Comparison of the frequency of Th17.1 cells and proportion of Tregs between the BALF and peripheral blood in sarcoidosis (*n* = 20) and IPF (*n* = 9) patients. **(A, B)** Flow cytometry dot plot of Th17.1 cells gated by the expression of CXCR3 and CCR6 in CCR4^–^ T cells from BALF and peripheral blood of a sarcoidosis patient, respectively. **(C)** There was a significant difference in the percentage of Th17.1 cells found in the BALF and peripheral blood in sarcoidosis patients (26.2% ± 4.2% vs. 2.1% ± 0.4%; *p *< 0.0001). **(D)** We found that sarcoidosis patients had a similar proportion of Tregs in their BALF and peripheral blood. **(E)** The ratio of Th17.1 cells to total Tregs of sarcoidosis patients was higher in BALF than in peripheral blood (6.6 ± 1.7 vs. 0.3 ± 0.1; *p*=0.001). A paired Student’s *t*-test was conducted for the paired comparison. **p < 0.01; ****p < 0.0001; ns, no significance. BALF, bronchoalveolar lavage fluid; PBMC, peripheral blood mononuclear cell; Sar, sarcoidosis; IPF, idiopathic pulmonary fibrosis; Tregs, regulatory T cells; Th17.1, T helper type 17.1.

It was interesting to note that the distribution frequency of Th17.1 cells was remarkably high in the BALF of sarcoidosis patients, whereas it was barely detectable in their peripheral blood (**Figures 5A, B**). The proportion of Th17.1 cells was much higher in the BALF than in the peripheral blood of sarcoidosis patients (*p *< 0.0001) (**Figure 5C**). However, there was no difference in the percentage of Tregs found in the BALF and blood of sarcoidosis patients (**Figure 5D**). Consequently, the ratio of Th17.1 cells to total Tregs in BALF was markedly increased, compared with the peripheral blood of sarcoidosis patients (*p *= 0.001) (**Figure 5E**). Both the increased proportion of Th17.1 cells in lungs and the higher ratio of Th17.1 cells to total Tregs in the BALF were observed only in sarcoidosis patients and not in IPF patients (**Figures 5C, E**). In contrast, a significantly higher proportion of Treg cells was observed in the BALF than the blood of IPF patients, but this was not the case for sarcoidosis patients (*p *= 0.005) (**Figure 5D**).

### Decreased fraction of circulating Tregs by immunoregulatory therapy for sarcoidosis patients

**Figure 6:**
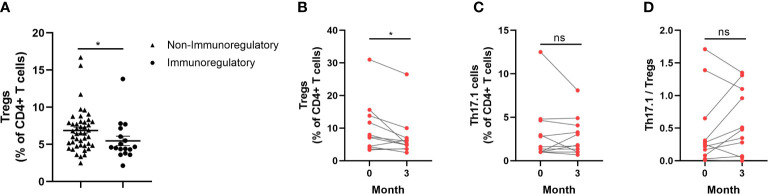
Changes of circulating Tregs proportion by immunoregulatory therapy for sarcoidosis patients. **(A)** Comparison of circulating Tregs proportion in patients who were taking with those not taking immunoregulatory medication (median 4.6% vs. 6.5%; *p *= 0.019). **(B)** The proportion of Tregs reduced so as to be different to a statistically significant degree 3 months after study enrolment in patients treated with glucocorticoids when compared with that measured at the baseline visit (median 5.1% vs. 7.3%; *p *= 0.022). **(C, D)** There was no difference in the proportion of Th17.1 cells and the ratio of Th17.1 cells to total Tregs at the 3-month follow-up visit, compared with the measurement at the baseline visit. An unpaired Student’s *t*-test was used to compare the proportion of circulating Tregs in patients who were taking immunoregulatory medication with those who were not. A paired Student’s t-test was conducted in the period between the baseline and follow-up visits. *p < 0.05; ns, no significance.

A total of 17 sarcoidosis patients had been taking immunoregulatory medication 3 months prior to their enrolment in the study, and they had lower proportions of circulating Tregs than those who had not been taking immunoregulatory medication (*p *= 0.019) (**Figure 6A**). In total, 11 sarcoidosis patients, who took glucocorticoids, were followed up for 3 months after the study. The distribution frequency of the circulating Tregs of these patients at the 3-month follow-up visit decreased significantly, compared with the baseline visit (*p *= 0.022) (**Figure 6B**). As for the proportion of Th17.1 cells and the ratio of Th17.1 cells to total Tregs, there was no difference when comparing those patients measured at the 3-month follow-up visit with those measured at the baseline visit (*p *= 0.513; *p *= 0.262) (**Figures 6C, D**).

## Discussion

The results of this study on the distribution of CD4^+^ T-cell subpopulations revealed that there was an increased frequency of Tregs in the blood, and of Th17.1 cells in the BALF, of sarcoidosis patients. This increased frequency of circulating Tregs was associated with disease activity and a fibrotic radiographic phenotype.

Tregs exhibit strong immunosuppressive capacities on various immune cells. The role that Tregs play in sarcoidosis remains controversial. Early reports claimed that the frequency of FOXP3-expressing blood Tregs was decreased in sarcoidosis patients compared with control participants (21). The expression of FOXP3, however, was markedly increased after corticosteroid medication (25). The majority of the studies in this field have found that an increased frequency of Tregs is usually observed in the active phase of this disease (23, 24), and our results are in line with this. Miyara et al. (23) found that CD4^+^ CD25^bright^ FOXP3^+^ Tregs accumulated in the peripheral blood and that there was a reduction in the frequency of circulating Tregs on resolution of sarcoidosis. The same research group showed, beside the amplification of blood Tregs in active sarcoidosis, that CD45RA^–^ FOXP3^BRIGHT^ Tregs proliferated and accumulated with granulomas (24). They found no correlation between the percentage of Tregs and sACE level, but a positive association to the extent of renal interstitial fibrosis (24). In patients with pulmonary sarcoidosis, an increased proportion of Tregs at the time of diagnosis was observed in patients developing chronic disease during follow-up (32). In our study, we found that there was a correlation between the proportion of circulating Tregs, sACE, and the CD4^+^/CD8^+^ cell ratio in the BALF, and patients with a fibrotic radiographic stage had higher proportion of circulating Tregs. We also showed in our study that sarcoidosis patients who had been taking immunoregulatory medication 3 months prior to study enrolment had a lower frequency of circulating Tregs than those who had not been taking such medication. Moreover, we showed that the frequency of circulating Tregs in sarcoidosis patients who were treated with corticosteroids after diagnosis was significantly decreased at the 3-month follow-up.

Despite their being present in large numbers, Tregs from sarcoidosis patients have been found to be functionally incompetent (33). Sarcoidosis patient-derived Tregs demonstrated a weaker capacity for inhibiting inflammatory cytokine production than Tregs obtained from healthy donors, and Tregs regained their suppressive capacity with disease resolution (34). Broos et al. (32) found that circulating Tregs were apoptotic-prone owing to their overexpression of CD95, leading to their impaired survival. In another study, it was reported that the repressor activity was impaired only in BALF Tregs and not in the peripheral blood Tregs of sarcoidosis patients, taking telomere length and the ability to produce transforming growth factor beta (TGF-β) and IL-10 into account (25). A functional analysis of Tregs was not conducted in our study, and this is an important limitation of our research.

A minority of sarcoidosis patients will develop chronic, and some even progressive disease, complicated by pulmonary hypertension or pulmonary fibrosis (3). There are some similarities between fibrotic sarcoidosis and IPF, the prototype of a progressive fibrotic interstitial lung disease (ILD). In this study, patients with IPF were used as disease controls. We found that there was a significantly higher frequency of circulating Tregs in active or fibrotic sarcoidosis patients than in patients with IPF. Regrettably, few fibrotic sarcoidosis patients donated BALF cells, as the majority of patients were either intolerant to or unwilling to undergo bronchoscopy, meaning that we were unable to compare the activity of lung Tregs in fibrotic patients with that in non-fibrotic sarcoidosis patients.

It has recently been recognized that Th17.1 cells play a role in the pathogenesis of sarcoidosis. Tøndell et al. (22) reported that the proportion of IFN-γ^+^ Th17.1 cells in BALF was higher in sarcoidosis patients than in healthy participants, and there was no significant difference between the proportion of these cells in sarcoidosis patients and in patients with other ILDs, including hypersensitivity pneumonitis (HP), IPF, connective tissue disease-associated ILD (CTD-ILD), and an unspecified ILD, and an increased ratio of lung Th17.1 cells to Tregs in sarcoidosis patients, compared with patients with other ILDs or healthy participants. Similarly, our study demonstrated that a significantly higher proportion of Th17.1 cells and an increased ratio of Th17.1 cells to Tregs in BALF were present in sarcoidosis patients compared with IPF patients. Ramstein et al. reported, in line with our data, that Th17.1 cells constituted approximately 30% of the lung CD4^+^ T cells and that their frequency was markedly higher in the BALF of sarcoidosis patients than in that of healthy volunteers (35). These authors identified Th17.1 cells, and not Th1 cells, as being the major producers of IFN-γ in the BALF of sarcoidosis patients (35). The proportion of lung Th17.1 cells at the time of diagnosis was higher in sarcoidosis patients who later developed chronic disease than in those who did not, supporting the idea that Th17.1 cells play a pathogenic role in promoting disease progression (20). In contrast, Kaiser et al. reported that a higher percentage of Th17.1 cells was correlated with a disease phenotype with a more favorable prognosis (19). To determine if Th17.1 cells play a pathogenic or protective role in the development of sarcoidosis, further investigation is needed.

The increased frequency of Th17.1 cells in sarcoidosis patients is apparently localized to the involved organs, such as the lung, as reflected by BALF analysis, or the mediastinal lymph nodes (20), but is absent in the blood, indicating that the lung microenvironment is a crucial effect. In our study, we also observed that there was a pronounced proportion of lung Th17.1 cells in the BALF of sarcoidosis patients, but barely detected them in these patients’ blood. This imbalanced distribution of Th17.1 cells between peripheral blood and BALF was observed only in sarcoidosis patients and not in patients with IPF, providing valuable information regarding immune cell compartmentalization to the lungs in various ILDs.

## Conclusions and future directions

In summary, we found that there was an increased proportion of Tregs in the peripheral blood, and of Th17.1 cells in the BALF, of sarcoidosis patients. An increased proportion of circulating Tregs was associated with disease activity and a fibrotic radiographic phenotype. The imbalanced distribution of Tregs and Th17.1 cells in the peripheral blood and BALF of sarcoidosis patients suggests that the lung microenvironment plays a significant role in the immunological pathogenesis of sarcoidosis. Functional studies on Tregs and Th17.1 cells are needed so that their exact roles, whether pathogenic or protective, in the immunological processes driving sarcoidosis can be uncovered.

## Data availability statement

The raw data supporting the conclusions of this article will be made available by the authors, without undue reservation.

## Ethics statement

The studies involving human participants were reviewed and approved by The Research Review Committee and the Ethical Review Committee of the China-Japan Friendship Hospital. The patients/participants provided their written informed consent to participate in this study.

## Author contributions

HZ designed the research route, collected the clinical information and biological samples, conducted the experiments, analyzed the data, and wrote the manuscript. DJ gave suggestions on the research route design and data analysis. LZ, GZ, and BX contributed to screening and enrolling patients and volunteers, data collection, and analysis. YC provided technical direction for experimental methods and revised the manuscript. UC revised the draft critically. HD contributed to the conception and design of the research, interpreted the data and results, and revised the manuscript. All authors contributed to the article and approved the submitted version.
